# Metabolic resilience: liraglutide’s potential in alleviating depressive symptoms

**DOI:** 10.1007/s11033-025-10641-w

**Published:** 2025-06-05

**Authors:** Omar Gammoh, Esam Qnais, Alaa A. A. Aljabali, Taher Hatahet, Abdelrahim Alqudah

**Affiliations:** 1 Department of Clinical Pharmacy and Pharmacy Practice, Faculty of Pharmacy, Yarmouk University, PO BOX 566, Irbid, 21163 Jordan; 2 Department of Biology and Biotechnology, Faculty of Science, The Hashemite University, Zarqa, Jordan; 3 Faculty of Pharmacy, Department of Pharmaceutics & Pharmaceutical Technology, Yarmouk University, Irbid, 21163 Jordan; 4 North Wales Medical School, Bangor University, Brigantia Building, Penrallt Road, Bangor, Gwynedd, Wales, LL57 2AS UK; 5 Department of Clinical Pharmacy and Pharmacy Practice, Faculty of Pharmaceutical Sciences, The Hashemite University, Zarqa, Jordan

**Keywords:** Liraglutide, Depression, Glucagon-like peptide-1 receptor agonist, Metabolic health, Neurobiological mechanisms, Clinical trials

## Abstract

Research in psychiatry requires substantial resources and interdisciplinary collaboration. The investigation of liraglutide’s potential to reduce depressive symptoms is a pioneering and novel approach. that ventures into underexplored mechanisms bridging metabolic and psychiatric domains. Originally approved for the management of type 2 diabetes, it has increasingly emerged as a potential therapeutic candidate in the complex landscape of mental health disorders. being examined for its ability to modulate depressive symptomatology, acting as a glucagon-like peptide-1 (GLP-1) receptor agonist. However, its action extends beyond traditional monoaminergic pathways, also influencing neuroplasticity, synaptic remodeling, and neuroinflammatory processes. Recent studies have shown preclinical and early-phase clinical insights into how liraglutide modulates mood-related neural circuits. These findings suggest mechanistic distinctions from conventional antidepressant pharmacotherapies. This manuscript presents a research gap. Specifically, it addresses gaps in both mechanistic understanding and translational potential, where liraglutide’s dual impact bridges the traditional divide between psychiatric and metabolic medicine. Liraglutide has demonstrated benefits in improving both glycemic control and depressive symptoms. These integrated effects position it as a candidate for dual-purpose interventions in patients with comorbid metabolic and psychiatric disorders. Scientists have shown details of how liraglutide affects depression. Emerging evidence remains preliminary yet promising, encouraging researchers to explore, question, and refine current psychiatric treatment models. In an era prioritizing biologically integrated therapeutics, liraglutide exemplifies the evolution of psychiatric drug development. In a field where innovation is key, liraglutide is a testament to evolving science. It provides a model for how metabolic agents may contribute to the future landscape of mental health therapeutics.

## Introduction

Recently, there has been a renewed focus on elucidating the intricate interplay between metabolic disorders and mental health, providing a more integrated framework for understanding systemic physiological regulation. Liraglutide, originally developed as glucagon-like peptide-1 (GLP-1) receptor agonist, was designed to address dysregulation in metabolic homeostasis [1, 2]. GLP-1, an endogenous incretin hormone, is secreted from enteroendocrine L cells, predominantly located in the distal part of the ileum and proximal colon, in response to postprandial glucose and carbohydrate intake, while present at lower levels during fasting. GLP-1 receptors, classified as members of the G-protein-coupled receptor (GPCR) superfamily, are primarily expressed in the pancreas, central nervous system, and gastrointestinal tract, with more limited expression in the heart, kidneys, vasculature, and peripheral nervous system [3]. Upon GLP-1 exerts its effects on pancreatic β-cells, inducing glucose-dependent insulin secretion via elevation of intracellular cyclic adenosine monophosphate (cAMP) concentrations [4]. Additionally, it promotes the survival and proliferation of pancreatic β-cells [5, 6]. GLP-1 also inhibits glucagon release from pancreatic α-cells, leading to reduced hepatic glucose output [7].

Liraglutide (also known commercially as Saxenda^®^, Victoza^®^, and Xultophy^®^) has transcended its original purpose as a GLP-1 receptor analog, initially developed for the treatment of type 2 diabetes mellitus and the prevention of diabetes-related cardiovascular complications [8–10]. Recent investigations have proposed a mechanistic linkage between GLP-1 signaling and mood regulation, thereby suggesting a potential connection between liraglutide and the pathophysiology of depressive symptoms. This positions liraglutide as a promising candidate in the emerging interface of metabolic and psychiatric medicine [11]. As researchers and clinicians continue to investigate novel therapeutic avenues for addressing mood disorders, liraglutide has emerged as a therapeutically relevant compound, with observed benefits extending beyond glycemic regulation into domains of neuropsychological health [12, 13].

**Fig. 1:**
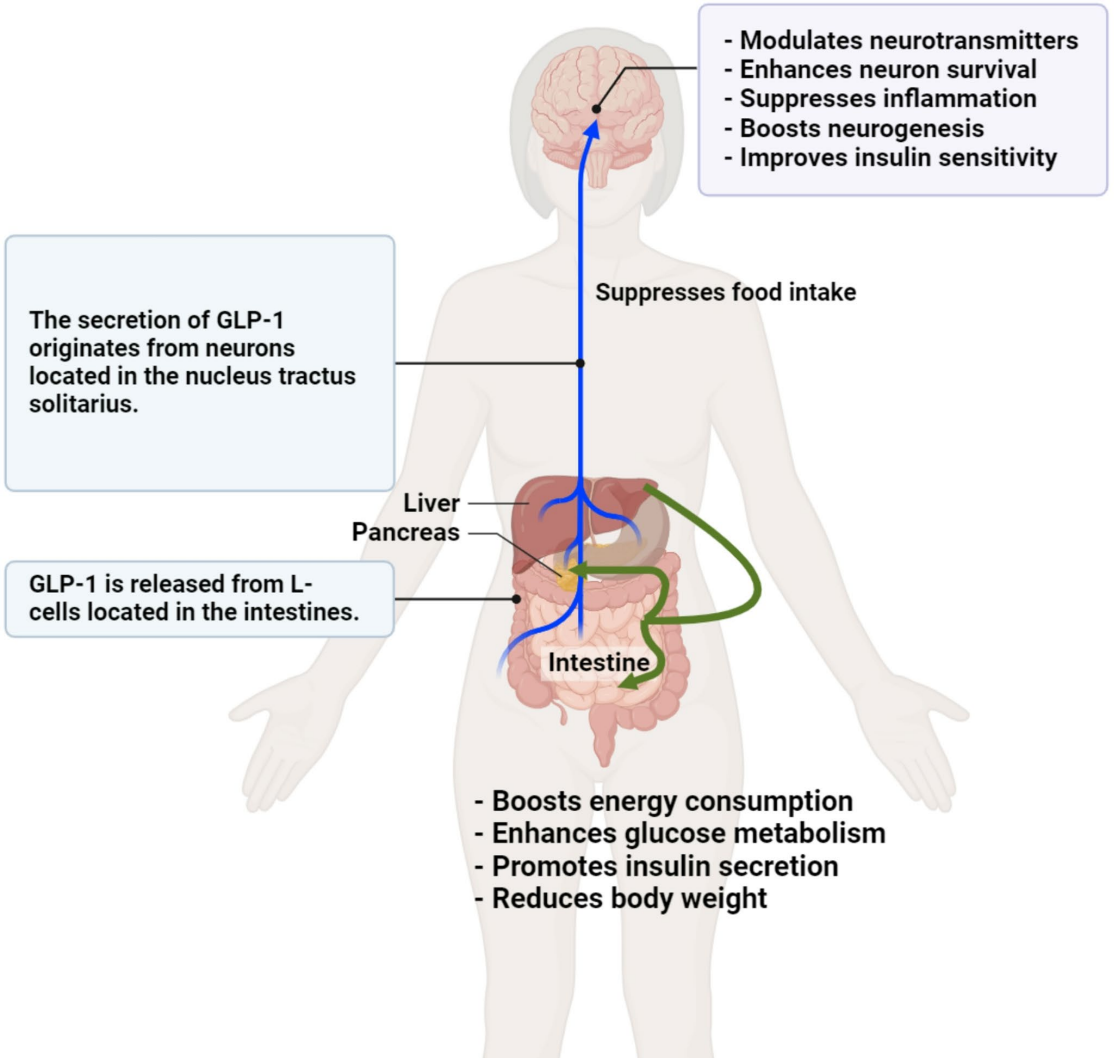
Liraglutide exerts its influence through multifaceted mechanisms, including neurotransmission modulation, neuronal survival enhancement, inflammation suppression, neurogenesis improvement, insulin sensitivity enhancement, energy consumption boost, glucose metabolism enhancement, insulin secretion promotion, and body weight reduction. The pivotal role of GLP-1, released from intestinal L-cells and originating from neurons in the nucleus tractus solitarius, serves as a linchpin in mediating the diverse effects of liraglutide

This review aimed to elucidate the intricate effects of liraglutide on depressive symptoms by exploring its biological foundations and clinical applications. When administered, glucagon-like peptide-1 (GLP-1) and GLP-1 receptor agonists reach the cerebrospinal fluid (CSF) and brain in the central nervous system, as evidenced in rodent models [14]. Increased GLP-1 concentrations in the brain have been postulated to affect hippocampal function [15]. The maintenance of glucose-insulin metabolic homeostasis plays a pivotal role in the gut-brain axis, given that glucose serves as the primary energy source for the brain [16]. Key incretins such as GLP-1 and GIP contribute to appetite regulation by diminishing hunger and enhancing satiety [16]. Additionally, they play a role in regulating glucose homeostasis by reducing blood glucose levels and modulating insulin release [17]. Figure 1 summarizes the role of GLP-1 in neurotransmission.

### Liraglutide’s distinct role in combating depression

Liraglutide, which functions as a GLP-1 receptor agonist, exhibits considerable promise in alleviating depressive symptoms. Numerous preclinical and mechanistic studies have suggested that liraglutide may improve cognitive function and alleviate mood-related pathological changes. However, direct clinical evidence in human populations remains limited, and current insights primarily reflect theoretical and preclinical findings [18]. Behavioral studies in mouse models have further illuminated the impact of liraglutide on reducing depressive- and anxiety-like behaviors while enhancing cognition [19]. A systematic review and meta-analysis have suggested that GLP-1 receptor agonists, including liraglutide, may serve as potential treatments for depressive symptoms in humans [19]. While affirming the potential of liraglutide in the treatment of depression, these findings underscore the need for additional research and clinical trials to establish its efficacy and safety in human patients [18].

Activation of hippocampal GLP-1 receptors via mitogen-activated protein kinases (MAPKs) enhances learning and memory [20]. GLP-1 also plays a role in enhancing hippocampal synaptic plasticity and synapse formation [20]. These findings provide evidence of GLP-1’s potential to generate new neurons, which can enhance cognitive function and reduce depressive behavior. Further research is required to understand the precise mechanisms by which GLP-1 influences the generation of new neuronal cells [21]. The ability to improve learning and memory, reduce inflammation, decrease apoptosis, modulate reward behavior, and reduce food intake are some of the functions that have been proven for GLP-1. GLP-1 and its analogs have been shown to offer future promise in the treatment of many diseases associated with cognitive impairment, including Alzheimer’s disease [22, 23]. The availability of liraglutide therapy for the treatment of depression is a novel advancement in a vast area of psychiatric studies. Traditionally accepted for the treatment of type 2 diabetes, liraglutide is emerging as a new beacon for mental health therapeutics [24].

Semaglutide, another GLP-1 receptor agonist with an extended half-life and similar central mechanisms, also crosses the blood-brain barrier and exhibits neuroprotective properties. Preliminary data suggest that semaglutide may exert antidepressant effects via modulation of neuroinflammation and hippocampal plasticity [25]. Although the current review centers on liraglutide due to its broader psychiatric characterization in preclinical and clinical settings, future comparative analyses that incorporate semaglutide are essential to delineate the nuanced roles of long-acting GLP-1 analogues in mood regulation.

Major depressive disorder (MDD) is a common mood disorder characterized by chronic painful symptoms of ruminative thoughts, impaired cognition, anhedonia, and deficiencies in attentional control [26, 27]. Depression is a complicated, multifaceted ailment that arises from a combination of genetic vulnerability and exposure to environmental stressors [28]. In addition to the personal toll, MDD significantly impacts family dynamics and contributes to substantial societal and healthcare costs [26, 27]. Beyond individual suffering, depression significantly diminishes the quality of life of patients and their families, imposing considerable economic and mental health burdens [27]. Research indicates that over 50% of those grappling with depression face chronic and recurrent challenges, with symptoms escalating to the risk of suicide without timely intervention [18]. This underscores the urgent need for timely diagnosis and intervention, reinforcing the importance of developing more effective therapeutic options and preventive strategies.

Depression intricately intertwines with various neurobiological factors, including neuroinflammation, neurotransmitter imbalances,, increased permeability of the blood-brain barrier, deficits in neurogenesis, and synaptic dysfunction [28]. Studies reveal that individuals with depression display impaired neurogenesis, delayed neural growth, and reduced synaptic plasticity [29]. Brain regions such as the prefrontal cortex (PFC), amygdala, and hippocampus critical for emotional regulation, cognitive processing, and stress response exhibit functional disruptions in depressive states. Diminished activity in the PFC and hippocampus, alongside increased activation of the amygdala, reflects core neuropathological changes observed in major depressive disorder [30]. Understanding these neurobiological foundations is essential for guiding the development of more targeted therapeutic strategies and early preventive interventions [12].

What sets liraglutide apart is its novel approach to address depressive symptoms. Operating as a GLP-1 receptor agonist, it transcends conventional neurotransmitter modulation, engaging in the domains of neuroplasticity and synaptic function [31]. Evidence suggests that liraglutide regulates mood through distinct pathways not shared by traditional antidepressants [32]. The fact that hippocampal neural plasticity is induced by liraglutide treatment in a manner that correlates with the relief of depressive and anxiety-like behaviors [19] suggests its potential to exert antidepressant effects and enhance cognitive function. Moreover, short- and long-acting GLP-1 receptor agonists, including liraglutide, effectively pass through the blood-brain barrier,, indicating their capacity to act directly within the central nervous system [33].

The neurobiological pathways through which liraglutide acts in depression are yet to be identified, but the results imply that it modulates several pathways [27, 34]. The mechanisms by which liraglutide exerts its antidepressant effects demand rigorous clarification, particularly through well-designed clinical studies [35, 36]. Liraglutide’s potential spans from molecular targets to real-world clinical implications, challenging the traditional separation between psychiatric and metabolic medicine. Its ability to improve both glycemic control and depressive symptoms reflects a dual therapeutic potential, advancing an integrated approach for comorbid conditions [36]. The emerging evidence base surrounding liraglutide’s neuropsychiatric mechanisms underscores a compelling opportunity for future investigation, inviting researchers to refine existing paradigms of psychiatric care [9, 35, 37]. Therefore, in a field characterized by innovation, liraglutide represents the face change of scientific inquiry and an opening into the future when metabolic agents might be key players in defining the face of mental health therapeutics [38].

### Overview of liraglutide

**Fig. 2:**
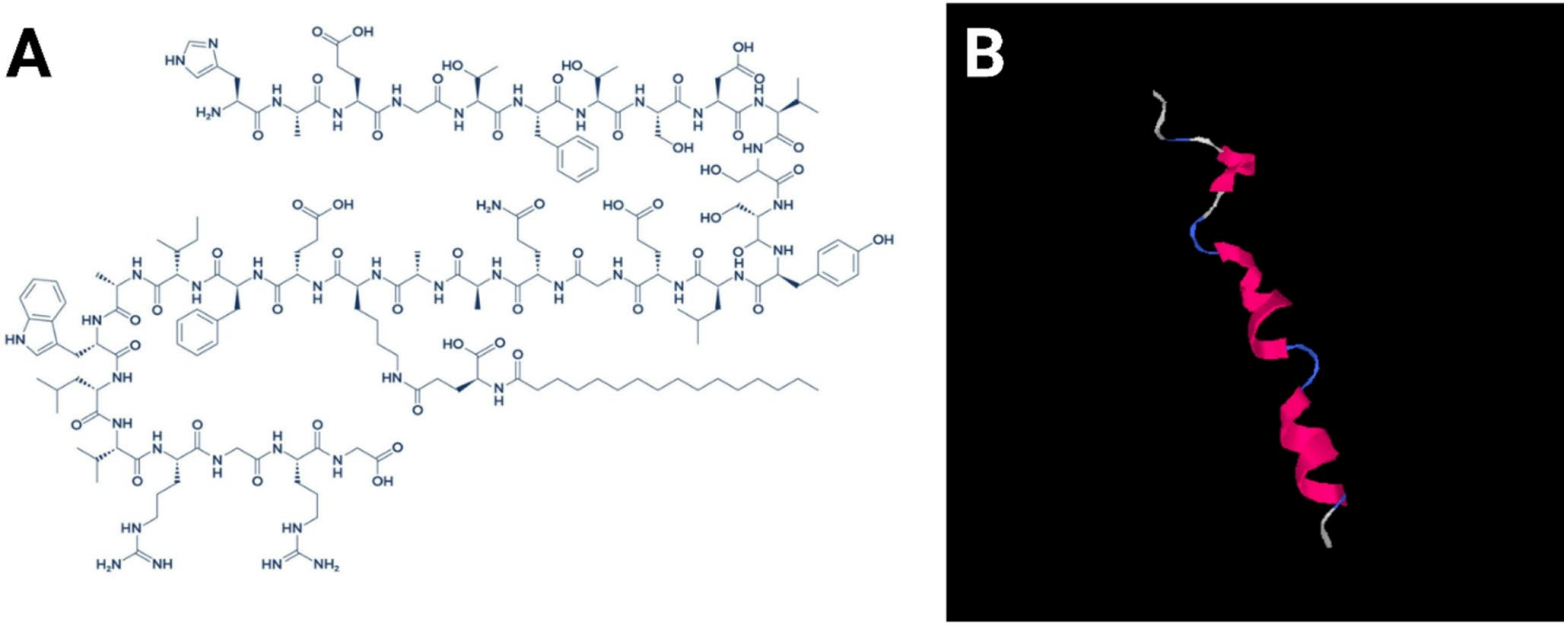
**A**) chemical structure of liraglutide (HAEGTFTSDVSSYLEGQAAKEEFIAWLVRGRG). **B**) schematic peptide illustration of liraglutide structure showing in pink the alpha-helix orientation. Image was generated by PyMol

Liraglutide, classified as a lipopeptide, serves as a human GLP-1 analog [8, 10, 21]. Its characteristic structural changes as shown in Fig. 2, include the substitution of lysine at position 27 with arginine and the linkage of a hexadecanoyl group to the remaining lysine through a glutamic acid spacer, determine its activity [20]. Liraglutide, in its major use, is used in combination with diet and exercise to help control glycemia in adults with type 2 diabetes mellitus [3, 9, 39]. Beyond its established metabolic applications, emerging evidence suggests that liraglutide may confer neuroprotective and mood-stabilizing effects. Understanding its pharmacological foundations, particularly the central mechanisms linked to its neuropsychiatric benefits, is increasingly recognized as essential [19].

The abundantly expressed GLP-1 receptor in the brain assumes a crucial role in modulating neurotransmission and synaptic plasticity [16, 29, 37]. Recent investigations have elucidated the impact of liraglutide on neuroplasticity, establishing a mechanistic basis for its putative antidepressant properties [1, 10, 18, 21, 40, 41]. Clinical trials not only reveal enhancements in glycemic control, but also clinically meaningful reductions in depressive symptoms, thereby prompting a re-evaluation of liraglutide’s therapeutic scope. This dual effect suggests liraglutide may represent a valuable adjunctive option in patients experiencing metabolic-psychiatric comorbidity [36, 42].

A comprehensive study on liraglutide exhibited significant analgesic effects in an extended murine model of osteoarthritis induced by sodium monoiodoacetate [43, 44]. This was also emphasized by the progressive recovery from mechanical allodynia over time when compared to the vehicle-treated group. Such findings, in which improvements in pain-related behavior were demonstrated in a sodium monoiodoacetate osteoarthritis mouse model, are due to the anti-inflammatory activity mediated by GLP-1 receptor activation, as observed after intra-articular administration of liraglutide [24, 45].

Liraglutide treatment also significantly suppressed the secretion of inflammatory mediators such as IL-6, PGE2, and nitric oxide [46]. A dose-dependent reduction in the expression of inflammatory genes was also observed in chondrocytes and macrophages [43]. It has also been shown that liraglutide can reverse the inflammatory in vitro polarized macrophage phenotype from the pro-inflammatory M1 phenotype to the anti-inflammatory M2 phenotype, indicating its anti-inflammatory potential [47]. In addition to its anti-inflammatory effects, liraglutide displays potent anti-catabolic activity by significantly diminishing the activities of metalloproteinases and aggrecanases, pivotal enzymes implicated in cartilage breakdown [39, 48]. Taken together, these findings suggest that liraglutide exhibits therapeutic potential in treating osteoarthritis, not only by mitigating pain, but also by reducing inflammation and safeguarding against cartilage degradation.

### Connection between metabolic disorders and mental health

The bidirectional interplay between metabolic disorders and mental health is a multifaceted line of scientific research that includes complex pathways and shared molecular cascades [49]. This interplay has revealed a multifactorial association between insulin resistance, inflammation, and neurotransmitter dysregulation as the focus of research. Recent comprehensive reviews, such as the work by Jones and Brown, offer mechanistic crosstalk between metabolic disturbances and depressive symptomatology; such reviews are important in providing foundational context for understanding the potential implications of liraglutide [50].

However, in the complex terrain of the potential of liraglutide to alleviate depressive symptoms, substantial progress has been made, yet critical questions remain unanswered. Neuroimaging techniques, specifically fMRI, can reveal the neural correlates underlying the antidepressant effects of liraglutide [47]. Knowledge of the sources of inter-individual variance in treatment responses and possible biomarkers is for advancing clinical translation. However, the view of liraglutide as a novel antidepressant is not entirely free of scientific controversy and debate [51].

Other researchers feel that the available evidence is still limited and urge cautious interpretation, emphasizing the need for large-scale well-controlled clinical trials. Such questions about the overlap of metabolic and antidepressant effects give rise to the potential problem of whether liraglutide’s mood-related effects are direct or secondary to metabolic improvements. However, answers to these concerns are imperative for defining the exact clinical niche and the population of patients most likely to benifit.

### Statement of the research question

However, the association between liraglutide use and depression remains unclear. Clarifying this relationship is essential for advancing evidence-based psychiatric applications of metabolic agents [38, 52]. Rather than being purely theoretical, this question is increasingly informed by emerging molecular evidence suggesting that liraglutide’s neurobiological targets may overlap with pathways implicated in mood regulation. Recent studies have provided a basis for further investigation as they have synthesized the results of many clinical trials. Despite encouraging leads, this field remains scientifically immature, complicated by conflicting data and methodological variability [53–56]. These studies emphasize a clear gap in understanding how liraglutide—originally approved for metabolic indications such as obesity might influence neuropsychiatric outcomes, particularly depression. Preliminary insights suggest a potential inverse association between anxiety symptoms and treatment adherence, though the underlying mechanisms remain undefined. Further mechanistic and clinical research is required to determine whether liraglutide exerts a direct therapeutic effect on depressive symptoms.

### Advantages and disadvantages

Liraglutide targets metabolic aberrations and may also alleviate depressive symptoms. However, like other pharmacological strategies, there are certain limitations. Its benefits, such as a relatively good side-effect profile and reported absence of weight gain, in particular, have been documented [36] the need to be weighed against several possible drawbacks, including the fact that it needs to be given by injection and challenges related to patient selection.

This updated section informs the reader of the scientific context for a review of the interface between metabolic illness and mental health, focusing on the therapeutic potential of liraglutide in the treatment of depressive symptoms. Building on the current discourse regarding the therapeutic potential of liraglutide for mental health, the current review critically engages with recent developments, controversies, and broader scientific debates [34, 37]. Thus, the repurposing of liraglutide from a metabolic agent to a potential adjunct in psychiatric care reflects the evolving landscape of psychopharmacology. The bio-psychosocial model remains a promising framework, but the interactions between underlying biological mechanisms and translational clinical applications must be carefully analyzed within an integrated therapeutic strategy for mental health. As the field progresses, unanswered questions underscore the need for rigorous interdisciplinary research to clarify the utility of liraglutide in mitigating depressive symptoms [23, 56].

### Justification

The scope of this review is to fill a critical gap in the literature by evaluating the available data on the relationship between liraglutide and depression. Although many studies have focused on the metabolic effects of liraglutide, its neuropsychiatric impact on depressive symptoms remains unclear. The current review, therefore, seeks to advance understanding by analyzing the potential mechanisms and existing preclinical and clinical evidence linking liraglutide to mood regulation. This evaluation aims to clarify whether liraglutide, known for its metabolic efficacy, could also contribute to enhancing mental health outcomes in patients with comorbid depressive symptoms.

This review is unique in that it provides coverage from both the metabolic and mental health perspectives to understand the effects of liraglutide. By integrating these dimensions, it offers a comprehensive framework for evaluating its therapeutic relevance. The present synthesis points to a new perspective in literature: beyond being a metabolic agent, liraglutide may represent a novel adjunctive strategy in the treatment of depression. This further characterizes the role of liraglutide in the evolving landscape of psychopharmacology and metabolic medicine, yielding new insights and highlighting directions for future clinical and translational research.

A more detailed SWOT analysis as shown in Table 1 revealed the potential advantages of liraglutide in the treatment of depressive symptoms, including the accompanying weaknesses and challenges that need to be considered. Opportunities for further research include the use of more advanced techniques, the inclusion of larger sample sizes across various populations, and adherence to the holistic treatment paradigm. Simultaneously, threats, including those associated with biases and individual variability, underline the need for methodological rigor and cautious interpretation of the results.

**Table 1:** SWOT analysis of the strengths, weaknesses, opportunities and threat in the potential use of liraglutide for the treatment of depressive symptoms

Strengths	Weaknesses
Biological Plausibility: Liraglutide, as a glucagon-like peptide-1 (GLP-1) receptor agonist, has demonstrated biological plausibility in modulating neurotransmitters, promoting neuroplasticity, and exerting anti-inflammatory effects, providing a strong scientific foundation.	Limited Dedicated Trials: The primary weakness lies in the scarcity of dedicated trials explicitly designed to investigate liraglutide’s psychotropic effects, relying predominantly on secondary analyses within trials primarily focused on metabolic endpoints.
Established Cardiovascular Benefits: Existing clinical trials, particularly the REWIND trial, have established the cardiovascular benefits of liraglutide, offering a solid starting point for exploring secondary endpoints, including depressive symptoms.	Methodological Constraints: The reliance on observational studies introduces methodological challenges, including confounding variables and an inability to establish causation, limiting the depth of evidence.
Real-World Applicability: Observational studies contribute real-world insights, showcasing liraglutide’s potential impact on depression in diverse patient populations beyond controlled clinical trials.	Heterogeneity in Study Populations: The predominantly diabetic study populations may limit the generalizability of findings to broader populations, raising questions about the specificity of liraglutide’s effects in individuals without metabolic disorders.
Holistic Approach: Liraglutide’s dual action in addressing metabolic disorders and potential neuropsychiatric effects positions it as a candidate for a holistic approach to mental health in individuals with comorbidities.	
Opportunities	**Threats**
Potential for Novel Treatment Paradigms: Liraglutide’s unique profile opens avenues for novel treatment paradigms that integrate metabolic and neuropsychiatric perspectives, potentially leading to more effective and personalized interventions.	Potential Type I Errors: Secondary analyses of trials, though insightful, may be susceptible to type I errors, emphasizing the need for dedicated trials designed explicitly for investigating the psychotropic effects of liraglutide.
Exploration of Neuroimaging Techniques: Future studies can leverage advanced neuroimaging techniques to unravel the neural correlates of liraglutide’s effects, offering a more in-depth understanding of its impact on brain structure and function.	Individual Variability: The potential for individual variability in response to liraglutide, optimal dosage, and sustainability of effects poses a threat to the generalizability and predictability of its impact on depressive symptoms.
Diverse Patient Populations: Opportunities exist for extending research beyond diabetic cohorts to include individuals without metabolic disorders, facilitating a comprehensive exploration of liraglutide’s impact across diverse patient populations.	Inherent Biases in Observational Studies: Observational studies, by nature, may be prone to biases, such as selection bias and unmeasured confounders, compromising the internal validity of findings.

### Liraglutide mechanism of action: detailed explanation of how liraglutide works

To understand the potential implications of liraglutide in the amelioration of depression, its complex mechanisms of action must be elucidated. Liraglutide belongs to a class of drugs known as GLP-1 receptor agonists as summarized in Table 2. The drug is a bioactive analog of the native endogenous hormone GLP-1, which is secreted by the gut in response to nutrient ingestion [48, 57, 58]. Endogenous GLP-1 acts in the body in such a way that it stimulates glucose homeostasis through increased insulin secretion and suppression of glucagon release from the pancreas [43].

**Table 2:** Mechanism of action of liraglutide

Mechanism of Action	Description
GLP-1 Receptor Agonism	Liraglutide is a long-acting glucagon-like peptide-1 (GLP-1) receptor agonist, binding and activating GLP-1 receptors. These receptors are primarily found in the pancreas and central nervous system. This activation enhances the effects of endogenous GLP-1.
Insulin Secretion	Liraglutide stimulates insulin secretion in a glucose-dependent manner. When blood glucose levels are elevated, it enhances the release of insulin from pancreatic beta cells, promoting glucose uptake by peripheral tissues and suppressing hepatic glucose production.
Glucagon Suppression	The drug suppresses the release of glucagon, a hormone that normally acts to increase blood glucose levels. By inhibiting glucagon secretion, liraglutide contributes to a reduction in blood glucose levels, especially in the postprandial state.
Delayed Gastric Emptying	Liraglutide slows down gastric emptying, leading to a more gradual absorption of nutrients from the digestive system. This effect helps regulate postprandial glucose levels and contributes to a feeling of fullness, aiding in weight management.
Central Nervous System Effects	Beyond its peripheral actions, liraglutide exerts effects on the central nervous system. It targets specific brain regions associated with appetite regulation and reward, influencing food intake and contributing to its weight management effects.
Neuroprotective and Neuroplastic Effects	Emerging research suggests potential neuroprotective and neuroplastic effects of liraglutide. It may impact brain structure and function, fostering neuroplasticity and potentially influencing mood regulation.
Anti-Inflammatory Properties	Liraglutide exhibits anti-inflammatory effects. It may mitigate inflammation associated with metabolic disorders and could have implications for conditions where inflammation plays a role, such as depressive symptoms.
Antioxidant Effects	The drug demonstrates antioxidant properties by scavenging free radicals. This action helps in reducing oxidative stress, which is implicated in various pathological processes, including neurodegeneration, and may contribute to its neuroprotective effects.

The added advantage of liraglutide is its long half-life due to acylation, which ensures the prolonged activation of GLP-1 receptors. When administered, it binds to these receptors in various tissues, including pancreatic islets, where it increases insulin secretion in a glucose-dependent manner, thereby improving glycemic control [59]. However, this mechanism is far beyond glucose control. The distribution of GLP-1 receptors in tissues is very wide, extending the evidence emerging for them in the brain, where they have been reported to influence mood and cognitive functions. Thus, activation of these receptors by liraglutide might extend the influence of neural circuits that modulate mood and possibly link its effect to an antidepressant effect [45].

### Discussion on its impact on metabolic pathways

The metabolic effects of liraglutide have been well-studied. In addition to stimulating insulin production, liraglutide is metabolized to enhance therapeutic effectiveness. Indeed, significant studies [13], have demonstrated that the drug can suppress appetite and reduce food intake, thereby promoting weight loss in individuals with obesity. This becomes particularly interesting because of the well-documented comorbidity between obesity, metabolic dysfunction, and depression.

In addition, liraglutide favorably influences lipid metabolism, leading to a drop in circulating triglycerides and the promotion of an anti-inflammatory milieu. Such metabolic improvements are relevant not only in the context of glycemic control but are also consistent with the general trend of recognizing the inextricable link between metabolic health and mental well-being [60].

### Overview of potential neurological effects

Liraglutide has the potential to symptomatically alleviate depressive symptomatology based on its mechanisms that affect neurological pathways. Recent neuroimaging studies have helped us understand the effects of liraglutide administration on the central nervous system [27]. These studies suggest that liraglutide may influence circuits governing emotional regulation and stress responses, although findings remain exploratory. It has been proposed that liraglutide promotes neuroplasticity and modulates key neurotransmitter systems, including serotonin and dopamine; however, the extent of these effects in the human brain remains uncertain due to limited permeability across the blood-brain barrier [61]. However, recent reports have also raised concerns regarding a possible association between GLP-1 receptor agonists and suicidal ideation. McIntyre (2024) hypothesized that such effects may stem from modulation of reward-related dopaminergic pathways, especially in vulnerable individuals, although robust human evidence is currently lacking [62].

However, caution should be exercised given the rapidly developing nature of this field. Although peripheral metabolic changes are well-documented, the blood-brain barrier remains a substantial obstacle, and the extent to which these changes translate into central nervous system modulation requires further clarification [63]. Mechanistic research on liraglutide has revealed a multifaceted spectrum of effects that surpass its role in glycemic regulation. Its interaction with metabolic pathways and potential neurological effects provide a compelling rationale for investigation of its role in the intricate landscape of depressive symptoms. The remaining part of the review examines empirical evidence and highlights both promises and challenges n positioning liraglutide as a potential adjunctive therapy in mental health care [54].

### Literature review on liraglutide and metabolic disorders

#### Studies showcasing the efficacy of liraglutide in treating metabolic disorders

There is strong and consistent evidence supporting the therapeutic role of liraglutide in managing metabolic disorders. Some clinical trials attesting to this effect include the LEADER trial [36, 64], which demonstrated the cardiovascular benefits of liraglutide in type 2 diabetes. Apart from its role in glycemic regulation, these studies have emphasized the role of liraglutide in the reduction of cardiovascular events. Furthermore, in recent years, pooled results from multiple large-scale trials have uniformly emphasized the contribution of liraglutide in terms of weight loss to insulin sensitivity and overall metabolic improvement as shown in Table 3. These systematic reductions in HbA1c levels and improvements in lipid profiles have rendered this class of drugs the cornerstone in the management of metabolic disorders [64, 65].

**Table 3:** Summary of the key clinical trials on the evaluation of liraglutide and other GLP-1 receptor agonists

Study Title	Study Design	Participants	Intervention	Outcome Measures	Key Findings
LEADER Trial: Liraglutide Effect and Action in Diabetes	Randomized Controlled Trial (RCT)	Type 2 Diabetes Patients at High Cardiovascular Risk	Liraglutide vs. Placebo	Primary Outcome: Composite of Cardiovascular Death, Nonfatal Myocardial Infarction, and Nonfatal Stroke. Secondary Outcomes: Multiple Cardiovascular and Renal Endpoints	Demonstrated significant reduction in the primary composite cardiovascular endpoint, establishing the cardiovascular safety of liraglutide. Additionally, showed improved glycemic control and reduced risk of kidney events.
SCALE Obesity and Prediabetes Trials	Randomized Controlled Trials (RCTs)	Obese or Overweight Individuals with or without Diabetes	Liraglutide vs. Placebo in Conjunction with Lifestyle Changes	Weight Loss, Glycemic Control, and Cardiovascular Risk Factors	Showed significant weight loss with liraglutide treatment, supporting its efficacy as an anti-obesity agent. Improved glycemic control and cardiovascular risk factors were also observed.
SUSTAIN Trials: Semaglutide Unabated Sustainability in Treatment of Type 2 Diabetes	Randomized Controlled Trials (RCTs)	Type 2 Diabetes Patients with Various Baseline Characteristics	Semaglutide (a GLP-1 analog like liraglutide) vs. Other Diabetes Medications	Various Glycemic and Cardiovascular Endpoints	Highlighted the effectiveness of semaglutide (like liraglutide) in achieving and maintaining glycemic control. Demonstrated cardiovascular safety and potential cardiovascular benefits.
DURATION Trials: Diabetes Therapy Utilization: Researching Changes in A1C, Weight, and Other Factors Through Intervention with Exenatide Once Weekly	Randomized Controlled Trials (RCTs)	Type 2 Diabetes Patients Inadequately Controlled on Other Medications	Exenatide Once Weekly (a GLP-1 analog) vs. Other Diabetes Medications	Glycemic Control, Weight Loss, and Cardiovascular Risk Factors	Showed sustained glycemic control and weight loss with exenatide once weekly, indicating the potential benefits of GLP-1 receptor agonists, including liraglutide, in managing type 2 diabetes.
Liraglutide and Cardiovascular Outcomes in Type 2 Diabetes	Randomized Controlled Trial (RCT)	Type 2 Diabetes Patients at High Cardiovascular Risk	Liraglutide vs. Placebo	Cardiovascular Outcomes	Revealed cardiovascular safety with liraglutide and a significant reduction in major adverse cardiovascular events, providing crucial insights into the cardiovascular effects of liraglutide.
GRADE Study: Glycemia Reduction Approaches in Diabetes: A Comparative Effectiveness Study	Comparative Effectiveness Study	Type 2 Diabetes Patients	Liraglutide vs. Other Antidiabetic Medications	Glycemic Control and Cardiovascular Endpoints	Demonstrated the effectiveness of liraglutide in achieving glycemic control, with a favorable cardiovascular profile compared to other antidiabetic medications.

#### Insights into the possible indirect effects on mental health through metabolic regulation

The intricate interplay between metabolic health and mental well-being serves as a backdrop to understanding the potential indirect effects of liraglutide on mental health. Studies looking into the psychosocial dimensions of metabolic interventions, as demonstrated by recent investigations, suggest that the metabolic benefits of liraglutide may extend beyond the physical domain to influence psychological well-being [66]. Such interconnections support an integrative view of health, though direct causality between metabolic improvement and psychiatric outcomes remains unconfirmed [67, 68]. Given the largely theoretical nature of current evidence, future studies must focus on clinical validation of these neuropsychiatric pathways in well-characterized patient subgroups. Importantly, patients with serious mental illnesses, including major depressive disorder, face a significantly increased risk of cardiometabolic diseases, which contributes to a reduced life expectancy. Even in the absence of direct antidepressant effects, GLP-1 receptor agonists such as liraglutide may offer clinically meaningful improvements in physical health outcomes by addressing metabolic dysfunction in this population.

#### Any contrasting studies or opinions in literature

Although the overall discourse highlights the positive outcomes of liraglutide, scientific debate does not lack opposing studies and arguments. Some studies, best typified by the findings of this study, have opposed the universally conclusive benefits of liraglutide, particularly in certain subpopulations. The argument that supports the shift toward personalized medicine is that individual responses to liraglutide are determined by genetic, metabolic, and psychosocial factors [36]. In addition, there is a discussion of the possible influence of confounding variables on the relationship between liraglutide and mental health. It is extremely difficult to control variables such as lifestyle changes, co-medications, and patient adherence; therefore, results must be interpreted with caution. Although such general trends are appreciated, the literature must be viewed with a critical eye because of the heterogeneity in study populations and methodologies [69].

In conclusion, most of the literature on liraglutide and metabolic disorders supports its potential for its effectiveness in glycemic control and reduction of cardiovascular risk. This prompted us to examine the indirect effects on mental health further. As we wade through the literature, such evidence synthesis and consideration of diverse views will be pivotal in constructing a comprehensive understanding of liraglutide’s role in the intricate interplay between metabolic and mental health [70].

### Link between metabolic health and mental health

#### Overview of existing literature linking metabolic disorders and depression

The deep interweaving of metabolic disorders with common disorders such as depression is a subject of contemporary scientific debate. Numerous epidemiological studies have consistently disclosed a two-way relationship between metabolic disorders, such as obesity, diabetes, and metabolic syndrome, and the prevalence of depressive symptoms [22, 70].

Hence, these studies suggest that individuals with depressive disorders is at high risk of developing a metabolic disorder, whereas hose experiencing metabolic irregularities is likely to also have a depressive disorder. This bidirectional association reflects shared pathophysiological mechanisms rather than mere comorbid coincidence [58, 71].

#### Exploration of shared pathways and biological mechanisms

It is important to understand the common pathways and biological mechanisms that may underline the relationship between metabolic health and mental well-being. Several reviews have provided detailed mechanistic frameworks regarding how changes in metabolic homeostasis affect neural circuits implicated in mood regulation has been reported [72]. Commonly shared pathways include disorders of insulin signaling, altered glucose metabolism, and impaired mitochondrial function. Among these, insulin resistance, one of the hallmarks of most metabolic disorders, affects the brain through disruption of neurotransmitter synthesis, reduced synaptic plasticity, and altered neuroinflammatory signaling. Furthermore, gut-brain-axis dysfunction [73] provides insight into how the gastrointestinal and central nervous systems communicate bidirectionally, thus resulting in significant effects on mood and behavior [26].

### Discussion on the role of inflammation and oxidative stress

These two broad avenues of metabolic and mental health converge and are likely to be expressed through shared pathway involving inflammation and oxidative stress. Chronic low-grade inflammation is responsible for the pathogenesis of depression and contributes to the development of metabolic disorders, as shown in Table 4. A landmark meta-analysis highlighted elevated levels of C-reactive protein, as significantly associated with increased risk for depressive symptoms [74]. Another shared factor between metabolic disorders and depression is oxidative stress, which is described as a condition marked by an imbalance between free radicals and antioxidant defenses [75]. It has been argued that there is a vicious cycle and those results from bidirectional interactions between inflammation and oxidative stress.

**Table 4:** Role of inflammation and oxidative stress in metabolic disorders and glp-1 agonists’ therapeutic potential

Aspect	Overview
Inflammation and Oxidative Stress in Metabolic Disorders	In metabolic disorders, such as type 2 diabetes and obesity, chronic low-grade inflammation and increased oxidative stress play pivotal roles in disease progression. This dysregulation contributes to insulin resistance, impaired glucose metabolism, and systemic complications.
Inflammatory Pathways	Various inflammatory pathways are activated in metabolic disorders, involving cytokines (e.g., TNF-α, IL-6), adipokines, and immune cells. These pathways disrupt normal cellular signaling, contributing to insulin resistance and tissue damage.
Oxidative Stress Mechanisms	Elevated levels of reactive oxygen species (ROS) and impaired antioxidant defense systems characterize oxidative stress in metabolic disorders. Mitochondrial dysfunction, endoplasmic reticulum stress, and hyperglycemia contribute to ROS generation.
Interplay with Metabolic Health	Inflammation and oxidative stress create a detrimental feedback loop with metabolic health. Dysfunctional adipose tissue, insulin resistance, and impaired beta-cell function contribute to increased inflammatory and oxidative responses, exacerbating metabolic dysfunction.
Implications for Neurological Health	Emerging evidence suggests a connection between systemic inflammation, oxidative stress, and neurological health. Inflammatory mediators and oxidative damage may impact the central nervous system, contributing to neurodegeneration and mood disorders.
Role of GLP-1 Agonists like Liraglutide	GLP-1 agonists, including liraglutide, exhibit anti-inflammatory and antioxidant properties. They may mitigate inflammation and oxidative stress by modulating immune responses, improving mitochondrial function, and reducing ROS production.
Clinical and Preclinical Studies	Clinical and preclinical studies have demonstrated the potential of GLP-1 agonists in reducing inflammatory markers, improving endothelial function, and attenuating oxidative stress. These effects contribute to the cardiometabolic benefits observed with liraglutide.
Controversies and Debates	Some debates exist regarding the specific mechanisms through which GLP-1 agonists exert anti-inflammatory effects. Additionally, the translation of findings from preclinical studies to clinical outcomes requires further exploration and validation.
Future Directions for Research	Future research should focus on unraveling the precise mechanisms by which GLP-1 agonists modulate inflammation and oxidative stress. Additionally, exploring the impact on neurological health and investigating potential synergies with existing anti-inflammatory therapies is warranted.

Understanding the nature of these common pathways is paramount, given that liraglutide may have a beneficial effect on reducing depressive symptoms. It is hypothesized that the metabolic benefits of liraglutide are not limited to glycemic control but may extend to the regulation of inflammation and oxidative stress. Recent studies [75, 76] have noted the anti-inflammatory and antioxidative properties of liraglutide, helping, to establish a mechanistic connection between its metabolic actions and putative mental health benefits it may confer [77]. In summary, the link between metabolic health and mental well-being is increasingly supported by mechanistic and clinical data. The interactions between inflammation, oxidative stress, and interlinked signaling cascades offer a comprehensive framework for studying bidirectional relationships. However, the possible role of liraglutide in reducing immune-related pathways is an interesting research avenue to further investigate the relationship between metabolism and mental health [25, 78].

### Existing studies on liraglutide and depression

Recently, the fields of metabolic science and neuropsychiatry have overlapped increasingly in research on liraglutide, a glucagon-like peptide 1 (GLP-1) receptor agonist, and its potential therapeutic effect on depressive symptomatology in the field of mental health. This review examined the methodological approaches, sample sizes, and findings of studies on the effects of liraglutide on depression over the last decade. Herein, we aimed to provide a comprehensive evaluation of both the promises and challenges of this noteworthy therapeutic avenue [79, 80].

### Presentation of clinical trials and observational studies

Scientific research on the ability of liraglutide to reduce depressive symptoms is based on a heterogeneous mix of clinical trials and observational studies. A major contribution of this study is that of the REWIND trial, a large, randomized trial that mainly considers the impact of liraglutide on cardiovascular outcomes in patients with type 2 diabetes. Therefore, analyses relative to cardiovascular endpoints are the primary methods, whereas secondary analyses are performed for mood-related endpoints [57, 81]. This trial had an excellent sample size and is, therefore, a good starting point for exploring the psychotropic effects of liraglutide. Additional perspectives were provided by observational studies, which further expanded the story from a real-life perspective regarding the link between liraglutide use and depression. A built-in benefit of observational designs is the ability to capture long-term effects and diverse patient populations beyond the confinements of controlled clinical trials [81].

### Evaluation of methodologies and sample sizes

A critical aspect in evaluating the current evidence is the robustness of methodologies and sample sizes. One of the foundations for proving a causal relationship is the presence of clinical trials that employ rigorous randomized controlled designs. Nonetheless, some methodological aspects require close scrutiny particularly the duration of intervention and the instruments used for outcome assessment. The REWIND trial does provide a good substrate, but methodological issues in this trial raise uncertainties regarding which specific mechanisms liraglutide might work by and how long it would take before any effects on mood become evident [82]. Observational studies are meaningful because they have the potential to be applied in the real world though they inherently contend with confounding variables. The potential influence of confounding variables such as patient adherence, concurrent medications, and lifestyle modifications limits the interpretation of the results [52]. While observational data contribute to the growing body of evidence, they also highlight the limitations intrinsic to nonexperimental designs.

### Summary of findings– positive and negative outcomes

Pooling of findings from the literature must include both positive and negative OUTCOMES. Although the primary results of the REWIND trial concerned cardiovascular endpoints, researchers were able to present substantial secondary findings [82]. In post-hoc analyses, liraglutide appeared to be associated with liraglutide was associated with a possible reduction in depressive symptoms when compared with placebo, but the results were not statistically significant [64]. This glimpse of a potential psychotropic effect further emphasizes the need for dedicated studies on the effect of liraglutide on mood [83]. While the majority of findings support potential benefits of liraglutide on mood and cognition, occasional observational reports have noted adverse neuropsychiatric symptoms. However, these are rare, not consistently replicated, and no causal relationship has been established in human studies. An added layer of uncertainty arises from the interaction of confounding variables and the lack of a control group in observational designs, which warrants further exploration [84].

### Controversies and debates on liraglutide potentials

This is a continuation of the discussion on the possibility of using liraglutide to reduce depressive symptoms, which has been controversial and debated. Other researchers, however, argue that secondary analyses of the REWIND study, while intriguing, may be susceptible to type I errors and thus require additional research using trials specifically designed to study the psychotropic effects of liraglutide. In addition, the scientific community is currently disputing the mechanistic basis of the psychotropic potential of liraglutide. Cardiovascular benefits mediated through glycemic control and weight loss, although independent of mood regulation, highlight the necessity of a deeper exploration of shared biological pathways interfacing metabolic health and mental well-being [84–86]. Collectively, current findings highlight a promising intersection between metabolic regulation and neuropsychiatric outcomes that warrants further investigation [24]. The current literature is indicative, ranging from clinical trials to observational studies. Due to methodological nuances and differences in sample sizes, interpretation of results should be approached with caution. Navigating the evolving landscape of liraglutide in the context of depression, this review seeks to unravel complex scenarios, provide key critical insights, and contribute to the ongoing scientific discussion on the psychotropic potential of this metabolic agent.

### Potential mechanisms of Liraglutide’s impact on depression

#### Discussion on neurotransmitter modulation

Interest in the potential psychotropic properties of liraglutide centers on its effects on neurotransmitter systems, a very intricate landscape in which molecules related to mood regulation such as serotonin and dopamine play critical roles. Pioneering preclinical studies [87], have suggested novel mechanisms by which how liraglutide modulates serotonin levels in brain areas associated with mood disorders. Similar to conventional antidepressants, liraglutide, through the activation of brain GLP-1 receptors, increases the availability of serotonin at the synapse. This is in line with the serotonin hypothesis of depression, in which changes in serotonin neurotransmission are involved in the etiology of mood disorders. Furthermore, the effect of liraglutide on other central neurotransmitter systems, including the dopaminergic and glutamatergic pathways, broadens our understanding of the possible mechanisms underlying its central effects on mood [48].

#### Examination of neuroplasticity and neurogenesis

The emerging concepts of neuroplasticity and neurogenesis provide context for considering the activity of liraglutide in depression [48, 88]. Neuroplasticity refers to the brain’s capacity to reorganize synaptic connections in response to stimuli, while neurogenesis involves the formation of new neurons, particularly in regions associated with emotional regulation. For example, in animal studies, liraglutide has been shown to enhance neuroplasticity and neurogenesis, resulting in increased activity in areas that support emotional processing. Intriguingly, liraglutide promoted increased neurogenesis in the hippocampus and other brain areas responsible for mood regulation. This supports the notion that the effects of antidepressants span beyond neurotransmitter changes alone; they are also likely to be structural changes [89].

#### Consideration of anti-inflammatory and antioxidant effects

Growing evidence in the fields of neuroplasticity and neurogenesis provides a rudimentary framework through which the effects of liraglutide on depression can be postulated. The two major processes active in the brain are neural circuit flexibility and formation of new connections, called neuroplasticity and neurogenesis [90]. For instance, liraglutide showed that it may have increased neuroplasticity and neurogenesis in regions key to emotional processing. Liraglutide increases neurogenesis in the hippocampus, which is central to mood regulation This kind of potency to induce structural changes is a property that is in line with an understanding of the effects of antidepressants not only as involving changes in neurotransmitter modulation but as involving a process of structural adaptation [91, 92].

#### Synthesis and future directions

These potential mechanisms may clarify the complex and full interactions through which liraglutide mediates depression. Thus, the dynamic interplay between neurotransmitter modulation, neuroplasticity, anti-inflammatory responce, and antioxidant effects suggests that liraglutide could improve depressive symptoms at multiple levels [34]. However, these findings urge a warrant a prudent given the preliminary nature of the current evidence. In addition, individual variations in responses, the most effective dose for neuropsychiatric effects, and the duration of these effects remain open questions [93]. In summary, the possible effects of liraglutide on depression represent a promising area for future investigation. An interdisciplinary approach combining metabolic science and neuropsychiatry will be particularly important in the future to fully understand the effects of liraglutide on mood disorders.

Future investigations may benefit from stratifying participants based on metabolic status, particularly targeting individuals with metabolically linked depression (e.g., those with insulin resistance, type 2 diabetes, or metabolic syndrome). This approach could enhance the precision of outcome assessments, reduce heterogeneity in treatment response, and help identify patient subgroups most likely to benefit from GLP-1 receptor agonist therapy.

### Unanswered questions and research gap

#### Identification of gaps in current research

While there is a growing research interest in the potential of liraglutide to alleviate depressive symptoms, noticeable gaps exist in the current research landscape. The identification of these gaps is crucial for driving future work in a more complete and scientifically focused manner into the complex interplay between liraglutide and depression [24, 60, 72]. One major limitation is the paucity of independent clinical trials specifically designed to assess the psychotropic activity of liraglutide. Existing evidence is largely derived from secondary analyses, which are exploratory in nature and not intended to confirm efficacy. The absence of trials in which depression was the primary outcome has resulted in several unknown outcomes. Causality should be demonstrated by strict well-controlled studies using standardized depression rating scales with long follow-up periods [11, 26]. Another limitation to the generalizability of the current study is that it primarily focused on patients with type 2 diabetes, thus reducing variation in patient populations. Expanding the research to incorporate individuals without metabolic disorders would make the results more generalizable and would delineate specific effects in distinct cohorts [94].

#### Proposals for future studies and experimental designs

To overcome these flaws, great care must be taken in experiments designed to connect liraglutide with depression. Trials must be randomized, double-blinded, and controlled with depression as the primary outcome. These studies must be designed with placebo arms and standardized psychiatric evaluations based on validated depression scales. Variations in dosage and duration should also be systematically investigated to identify the optimal liraglutide regimen for producing clinically meaningful neuropsychiatric outcomes [95].

Longitudinal studies should be conducted to determine the trajectory of the effects of liraglutide on depressive symptoms. Such designs would facilitate the exploration of the persistence of the effects and allow the identification of potential differences in short- and long-term outcomes [57]. Furthermore, neuroimaging methods such as fMRI and PET scans may help identify the neural substrates underlying of the neural correlates of liraglutide effects. These techniques allow for direct observation of changes in brain activity and neurotransmitter signaling, offering mechanistic insights into liraglutide’s central actions [71, 72].

#### Consideration of diverse patient populations and comorbidities

Emphasis should be placed on including heterogeneous patient populations to investigate complex interactions between liraglutide and depression. Studies within the diabetes population have created the basis, but further research must expand to include individuals without metabolic disorders and those with varying different metabolic profiles. Differences in potential responses across heterogeneous populations may offer clinically relevant insights into the broad application of liraglutide [96]. Finally, the issue of comorbidities is also important. Depression seldom appears as an isolated disorder but often occurs as a symptom or comorbidity with other psychiatric or medical disorders. Future research should clarify the impact of liraglutide on patients with comorbidities, such as cardiovascular diseases or anxiety disorders in a more systematic manner. This would strengthen the framework of personalized medicine and opened a field of exploration for potential synergistic effects or contraindications [38, 73].

Therefore, research on liraglutide and depression is promising but requires precision and refinement to reach translational utility. This has advanced the identification of distinct gaps in current studies. Future work should include studies with meticulous experimental designs, diverse patient populations, and a full exploration of comorbidities. Through dedicated work by the scientific community, the field may come to a more detailed understanding of the potential of liraglutide in mental health [97].

### Challenges and limitations

#### Critical evaluation of limitations in existing studies

We must continue to make our way through the potential of liraglutide to ameliorate the symptoms of depression with a critical and candid perspective on the limitations of the current studies. Although promising, understanding these limitations is imperative regarding the implications of the findings and guiding research efforts [38]. One important limitation is that most of these were the results of secondary analyses in the frameworks of trials primarily designed for metabolic endpoints. For instance, the REWIND trial is primarily aimed at cardiovascular outcomes; therefore, secondary analyses of the depression domain might be limited in their reach, as it will be an exploratory approach. Therefore, one would question under such design limitations how robust and in-depth the data specific to depression would be [82, 83, 98]. However, this also introduces a set of methodological issues such as reliance on observational studies. Observational studies cannot establish causation by design and the results should be interpreted with caution. Confounding with non-observed variables will likely remain, making it difficult to attribute the observed associations to liraglutide alone [8, 45, 99].

#### Discussion on potential biases or confounding variables

Additionally, bias and confounding variables should be considered to refine the reliability of the conclusions drawn from this study. Potential sources of bias include heterogeneity among study populations with metabolic disorders characterized by comorbidities and lifestyle factors that may confound the effect of liraglutide on depressive symptoms [22, 31, 53]. Patient adherence is a critical variable that may influence treatment outcomes over time. Variability in adherence rates across individuals might affect outcomes and, lead to underestimation or overestimation of the effect of liraglutide on depression. This finding emphasizes the need for systematic monitoring of adherence and transparent reporting of adherence rates in future studies [9, 36, 42, 44].

#### Suggestions for improving study designs

The following key improvements in the study design are needed to overcome these hurdles and increase the strength of future research. Foremost among these is the emphasis on dedicated trials in which depression is the primary endpoint. Such trials need to be adequately powered and placebo-controlled, and standardized psychiatric assessments must to be incorporated to capture subtle changes in depressive symptoms [26, 33, 39]. Future studies that use advanced statistical methods, including propensity score matching can help reduce confounding variables in observational studies. For instance, prospective cohort studies with rigorously matched control groups would provide a more controlled setting for researching the relationship between liraglutide and depression [30, 36]. Improvements in data granularity are also essential. Future studies should systematically record information on potential confounders such as lifestyle factors, concurrent medications, and comorbidities to allow for appropriate adjustments in the analysis. Stratified analyses across these factors should also be considered to further enhance our understanding of the different responses across subpopulations. Therefore, although this is a preliminary step in opening the way to a potentially promising therapeutic avenue for liraglutide and depression, strict evaluation of the challenges and limitations is necessary to advance the current evidence base. Methodological limitations, biases, and confounding variables highlight the urgent need for improved methodological rigor [39, 73, 94, 95, 99]. The quality of evidence on the effects of liraglutide on depressive symptoms will improve if the scientific community adopts dedicated trials with more advanced statistical techniques and meticulous data collection [23, 53, 55, 78].

## Conclusion

In summary, the research on liraglutide’s ability to alleviate depression demonstrates a complex and multifaceted nature at the intersection of metabolic and neuropsychiatric sciences. This comprehensive review incorporates data from recent clinical trials, such as REWIND, and observational studies to provide an overview of the current state of scientific knowledge, methodologies, and potential mechanisms. The review begins with a thorough introduction that establishes a crucial link between metabolic disorders and mental health. Furthermore, the acknowledgment of study limitations, including secondary analyses and potential biases, adds a critical layer of evaluation necessary for contextualizing findings.

The studies examined in this context shed light on the intricate workings of liraglutide in depression, including its effects on neurotransmitters, neuroplasticity, and anti-inflammatory mechanisms. These findings not only offer a comprehensive overview of existing knowledge but also highlight the evolving complexity of liraglutide’s role in neuropsychiatry and metabolic medicine. The identified gaps and unanswered questions provide a thought-provoking challenge, urging a critical evaluation of the current state of the field and its future direction. The proposed studies for the future, which involve diverse populations and better experimental designs, emphasize the importance of conducting research in a rigorous and comprehensive manner. Although some challenges and limitations were not addressed, they were nonetheless recognized as an inherent part of scientific inquiry. An honest discussion of biases and confounding variables, as well as a consideration of improved study design possibilities, demonstrates a commitment to scientific rigor. This dedication to field development, while acknowledging uncertainties and encouraging ongoing dialogue among professionals, represents a significant step forward. The review sections of this work have not only deepened our understanding of liraglutide’s potential to reduce depressive symptoms, but also positioned it as a focal point for interdisciplinary exploration in mental health research. The reflective conclusion may serve as a call for active, not passive, engagement with this work: a plea for further investigation, inquiry, and collaboration in the pursuit of a better understanding of the complex interplay between metabolic and mental health.

## Data Availability

No datasets were generated or analysed during the current study.
