# Correction: Metabolic resilience: liraglutide’s potential in alleviating depressive symptoms

**DOI:** 10.1007/s11033-025-10965-7

**Published:** 2025-08-27

**Authors:** Omar Gammoh, Esam Qnais, Alaa A. A. Aljabali, Taher Hatahet, Abdelrahim Alqudah

**Affiliations:** 1 Department of Clinical Pharmacy and Pharmacy Practice, Faculty of Pharmacy, Yarmouk University, PO BOX 566, Irbid, 21163 Jordan; 2 Department of Biology and Biotechnology, Faculty of Science, The Hashemite University, Zarqa, Jordan; 3 Faculty of Pharmacy, Department of Pharmaceutics & Pharmaceutical Technology, Yarmouk University, Irbid, 21163 Jordan; 4 North Wales Medical School, Bangor University, Brigantia Building, Penrallt Road, Bangor, Gwynedd, Wales, LL57 2AS UK; 5 Department of Clinical Pharmacy and Pharmacy Practice, Faculty of Pharmaceutical Sciences, The Hashemite University, Zarqa, Jordan

**Correction to: Molecular Biology Reports (2025) 52:550** 10.1007/s11033-025-10641-w

In this article, the corresponding author's name Taher Hatahet was incorrectly written as Taher Hataet.

The original article has been corrected.

